# Prevalence and risk factors of bone metastasis and the development of bone metastatic prognostic classification system: a pan-cancer population study

**DOI:** 10.18632/aging.205224

**Published:** 2023-11-19

**Authors:** Zhouyang Hu, Sheng Yang, Zhipeng Xu, Xiaoling Zhang, Hong Wang, Guoxin Fan, Xiang Liao

**Affiliations:** 1 Department of Pain Medicine, Huazhong University of Science and Technology Union Shenzhen Hospital, Shenzhen, China; 2 Guangdong Key Laboratory for Biomedical Measurements and Ultrasound Imaging, National-Regional Key Technology Engineering Laboratory for Medical Ultrasound, School of Biomedical Engineering, Shenzhen University Medical School, Shenzhen, China; 3 Department of Orthopedics, Shanghai Tenth People’s Hospital, Tongji University School of Medicine, Shanghai, China; 4 Spinal Pain Research Institute, Tongji University School of Medicine, Shanghai, China

**Keywords:** pan-cancer analysis, bone metastasis, prevalence, risk factors, prognostic classification system

## Abstract

Background: The prevalence of bone metastasis (BM) varies among primary cancer patients, and it has a significant impact on prognosis. However, there is a lack of research in this area. This study aims to explore the clinical characteristics, prevalence, and risk factors, and to establish a prognostic classification system for pan-cancer patients with BM.

Methods: The data obtained from the Surveillance, Epidemiology and End Results database were investigated. The prevalence and prognosis of patients with BM were analyzed. Hierarchical clustering was used to develop a prognostic classification system.

Results: From 2010 to 2019, the prevalence of BM has increased by 41.43%. BM most commonly occurs in cancers that originate in the adrenal gland, lung and bronchus and overlapping lesion of digestive systems. Negative prognostic factors included older age, male sex, poorer grade, unmarried status, low income, non-metropolitan living, advanced tumor stages, previous chemotherapy, and synchronous liver, lung, and brain metastasis. Three categories with significantly different survival time were identified in the classification system.

Conclusions: The clinical features, prevalence, risk factors, and prognostic factors in pan-cancer patients with BM were investigated. A prognostic classification system was developed to provide survival information and aid physicians in selecting personalized treatment plans for patients with BM.

## INTRODUCTION

The epidemiology of cancer shows a continuous increase in the number of cases and related mortality. In 2023, it is projected that there will be over 1.9 million new cancer cases and more than 0.60 million deaths attributed to this deadly disease in the United States [1]. A nationwide population-based registry study has revealed that 66.7% of deaths related to solid tumors in cancer patients were due to metastasis over the past decade [2]. Bone metastasis (BM) occurs in approximately 70% of patients with prostate and breast cancers, while it is observed in about 30% to 40% of patients with lung cancer [3].

BM occurs due to various factors, these include the high blood flow found in red marrow, which facilitates the migration and establishment of tumor cells. Additionally, tumor cells possess adhesive molecules that bind them to stromal cells present in the bone marrow, facilitating their settlement and growth. Finally, the secretion of angiogenic and bone-resorbing factors by tumors enhances their growth and enables access to resorbed bone matrix for further proliferation, leading to the development and spread of metastatic tumors in bones [4–6]. BM commonly cause pain and can lead to significant morbidity, such as skeletal-related events, which results in a notable occupation of hospital resources [7]. However, most existing studies predominantly investigated the epidemiology, risk factors, and prognostic factors of bone metastasis originating from a specific primary cancer. Pan-cancer analysis helps identify commonalities, differences, and overarching patterns that may exist across different cancers that present with BM, leading to a better understanding of BM as a whole and potentially informing more effective prevention, diagnosis, and treatment strategies [8].

The considerable heterogeneity in BM across different types of cancer presents challenges in classifying cancer patients with bone metastases. However, prognostic-based patient classification is crucial in determining the optimal treatment approach. Hierarchical clustering is a robust and versatile technique used to analyze multiple factors and classify different cancers that develop BM based on their similarities and differences [9]. Therefore, establishing a prognosis-based classification system of cancers with BM can help guide doctors in the selection of more individualized treatment regimens for the best outcomes for patients.

Therefore, this study aimed to systematically describe the characteristics, prevalence, and establish a prognosis-based classification system of cancers with BM via using data from the Surveillance, Epidemiology, and End Results (SEER) database.

## RESULTS

### Baseline characteristics

There were 2,712,514 patients included in this study. For patients in the construction cohort (n = 1,546,109), the mean age was 62.4 ± 14.4 years, while 49.4% were male, 80.2% were white, 54.4% were married, 28.2% were with income larger than $7,5000 per year and 87.9% were metropolitan. The mean age for the validation group (n = 1,166,405), was 62.4 ± 14.4 years, with 49.3% being male, 78.0% white, 53.6% married, 36.0% having an annual income of more than $75,000 and 88.1% residing in metropolitan areas. Due to the relatively large sample size of the participants, differences of each parameter were shown in Table 1.

**Table 1:** Demographic and characteristics of patients with and without BM in the construction and validation cohorts.

**Items**	**Construction cohort**		***X^2^/Z***	***P***	**Validation cohort**		***X^2^/Z***	***P***
	**With BM**	**Without BM**			**With BM**	**Without BM**		
	**N (%)**	**N (%)**			**N (%)**	**N (%)**		
*Total*	75712 (5.1)	1470397 (95.1)			63610 (5.5)	1102795 (94.5)		
**Age**	62.4	14.4 (SD)	31.6	<0.01	62.8	14.4 (SD)	26.2	<0.01
<65	35216	787739			27569	564188		
≥65	40496	682658			36041	538607		
**Sex**			29.9	<0.01			27.4	<0.01
Male	43788	720029			37500	538036		
Female	31924	750368			564759	26110		
**Race**			3.4	0.0007			2.9	0.003
White	59677	1179968			49249	861045		
Black	9410	153251			7763	116233		
Asian or Pacific islander	5938	109039			5682	91069		
American native	529	9565			515	7610		
Unknown	158	18574			401	26838		
**Tumor grade**			71.7	<0.01			57.1	<0.01
Grade I	1742	189277			801	133960		
Grade II	9284	452543			4021	268231		
Grade III	22848	348756			9446	150371		
Grade IV	2890	65095			1234	36039		
Unknown	38948	414726			48108	514194		
**Marital status**			3.1	0.002			0.7	0.47
Married	38809	802152			32546	593066		
Unmarried	33240	127176			2880	101033		
Unknown	3663	541069			28184	408696		
**Income**			2.6	0.009			2.2	0.03
>75000$	20061	415556			21856	397950		
≤75000$	55648	1054630			41754	704771		
Unknown	3	211			0	74		
**Living area**			1.8	0.07			1.9	0.06
Metropolitan	65405	1293322			55092	972222		
Non-metropolitan	10192	175123			8417	129164		
Unknown	115	1952			101	1409		
**T stage**			41.2	<0.01			46.4	<0.01
T1	10335	622363			8312	455020		
T2	16352	353327			12103	237915		
T3	13920	246093			9266	167109		
T4	17890	106940			13611	76546		
Unknown	17215	141674			20318	166205		
**N stage**			40.8	<0.01			84.3	<0.01
N0	21770	1036349			16325	703701		
N1	15368	203442			12881	142407		
N2	18504	113557			11635	68750		
N3	9292	33039			7574	22696		
Unknown	10778	84010			15195	165241		
**Liver metastasis**			71.8	<0.01			78.5	<0.01
Yes	15233	46593			12566	35777		
No	44194	1251868			38854	920051		
Unknown	1440	1165			804	695		
**Lung metastasis**			90.4	<0.01			87.6	<0.01
Yes	15398	34146			12804	26521		
No	43314	1263056			38318	928691		
Unknown	2155	2424			1102	1311		
**Brain metastasis**			58.7	<0.01			44.7	<0.01
Yes	8908	16506			7236	11418		
No	50314	1282433			44073	944673		
Unknown	1645	687			915			
**Systemic therapy**			50.1	<0.01			84.5	<0.01
Yes	12218	444670			11303	352025		
No/ Unknown	63494	1025727			52307	750770		
**Radiotherapy**			53.0	<0.01			69.6	<0.01
Yes	31939	457651			24375	335081		
No/Unknown	43773	1012746			39235	767714		
**Chemotherapy**			38.2	<0.01			55.7	<0.01
Yes	38848	1034589			31724	764444		
No/Unknown	36864	435808			31886	338351		

**Figure 1:**
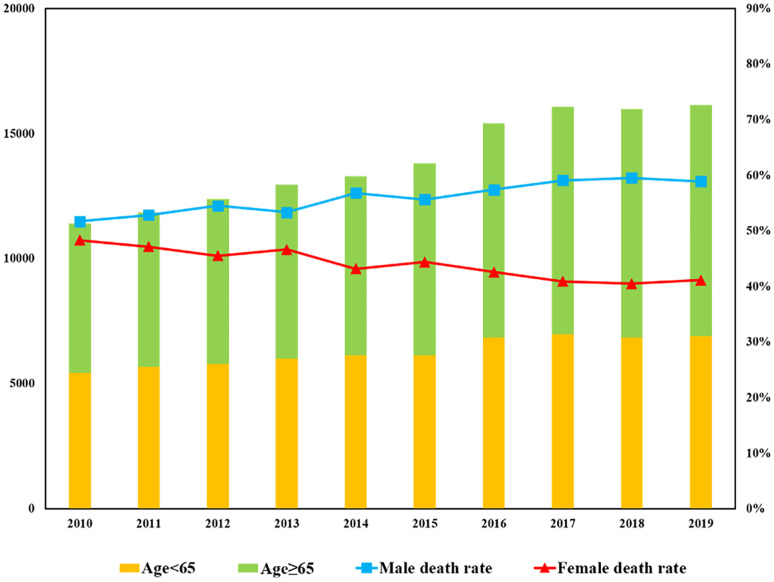
Annual trend of metastatic patients with BM.

**Figure 2:**
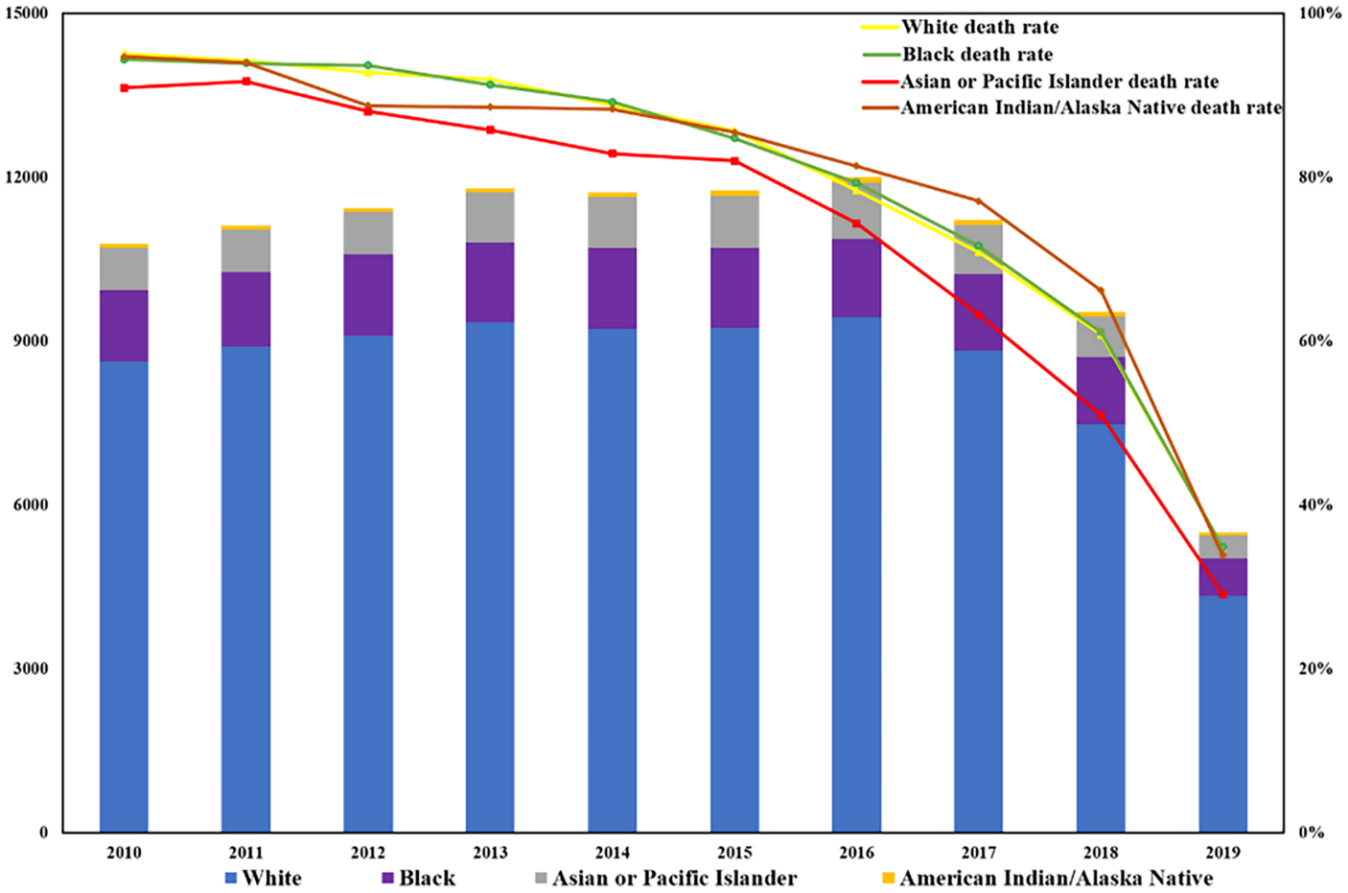
The state and annual trend of BM mortality among different races.

The number of patients with BM has been increasing annually, rising from 11,408 cases in 2010 to 16,138 cases in 2019 representing a significant overall increase of 41.43%. The proportion of those aged ≥ 65 has been growing significantly faster than those under 65. From 2010 to 2019, the proportion of people aged ≥ 65 increased from 52.4% to 57.2%, as shown in Figure 1. It can be observed that in patients with BM, the proportion of male patients has also increased considerably, from 51.7% to 58.9%, while the female proportion has decreased from 48.3% to 41.1%. In addition, for pan-cancer patients who were with BM, the death tolls among different races have shown an upward trend followed by a downward trend over the past decade (see Figure 2). However, the mortality rate in each race has decreased year by year, with the largest ethnic group being white, which has experienced a significant decrease from 95.1% in 2010 to 34.8% in 2019.

### Prevalence of bone metastasis

**Figure 3:**
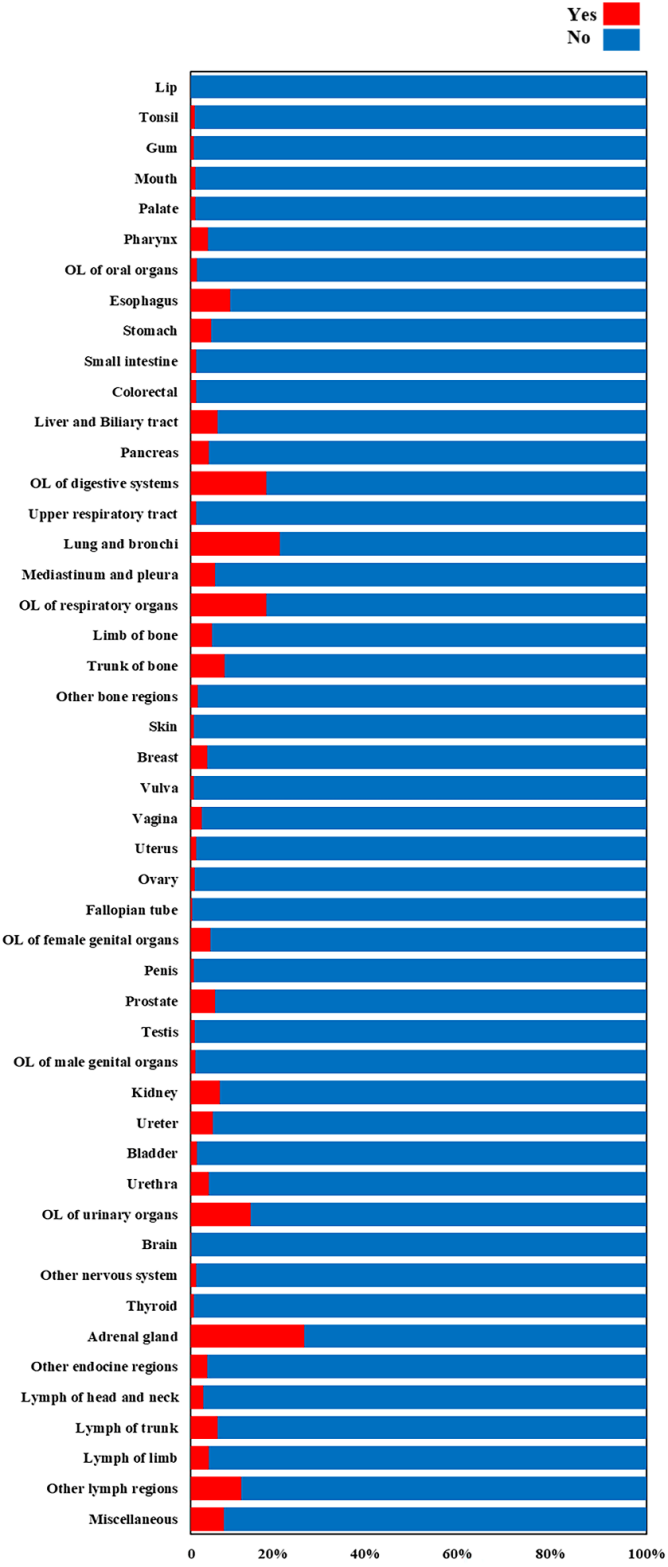
The prevalence of BM among different primary cancers.

The prevalence of BM varies greatly among different primary cancers (as shown in Figure 3). The five primary cancers with the lowest incidence of BM were lip cancer with 0.07%, brain cancer with 0.20%, fallopian tube cancer with 0.35%, and vulva and penis cancer with the same incidence rate of 0.71%. On the other hand, the top 5 cancer types that were most prone to BM were adrenal gland cancer, lung and bronchus cancer, cancers in overlapping lesion (OL) of digestive, respiratory, and other urinary organs, with prevalence rates of 25.03%, 19.69%, 16.73%, 16.67%, and 13.24%, respectively.

In both the construction and validation cohorts, significant differences were observed between patients with or without BM in terms of age, gender, race, marital status, average family income, tumor grade, T/N staging, and concomitant liver, bone, and lung metastases, as demonstrated in Table 1. Interestingly, no significant difference was found in the occurrence of BM between patients living in metropolitan (> 250 thousand population) and non-metropolitan areas. Furthermore, the prevalence of BM was higher among patients with concomitant liver, bone, or lung metastases compared to those without. Statistical differences were also noted in the number of patients with BM who received systemic treatment, radiotherapy, or chemotherapy.

### Risk factors to the bone metastasis occurrence

**Figure 4:**
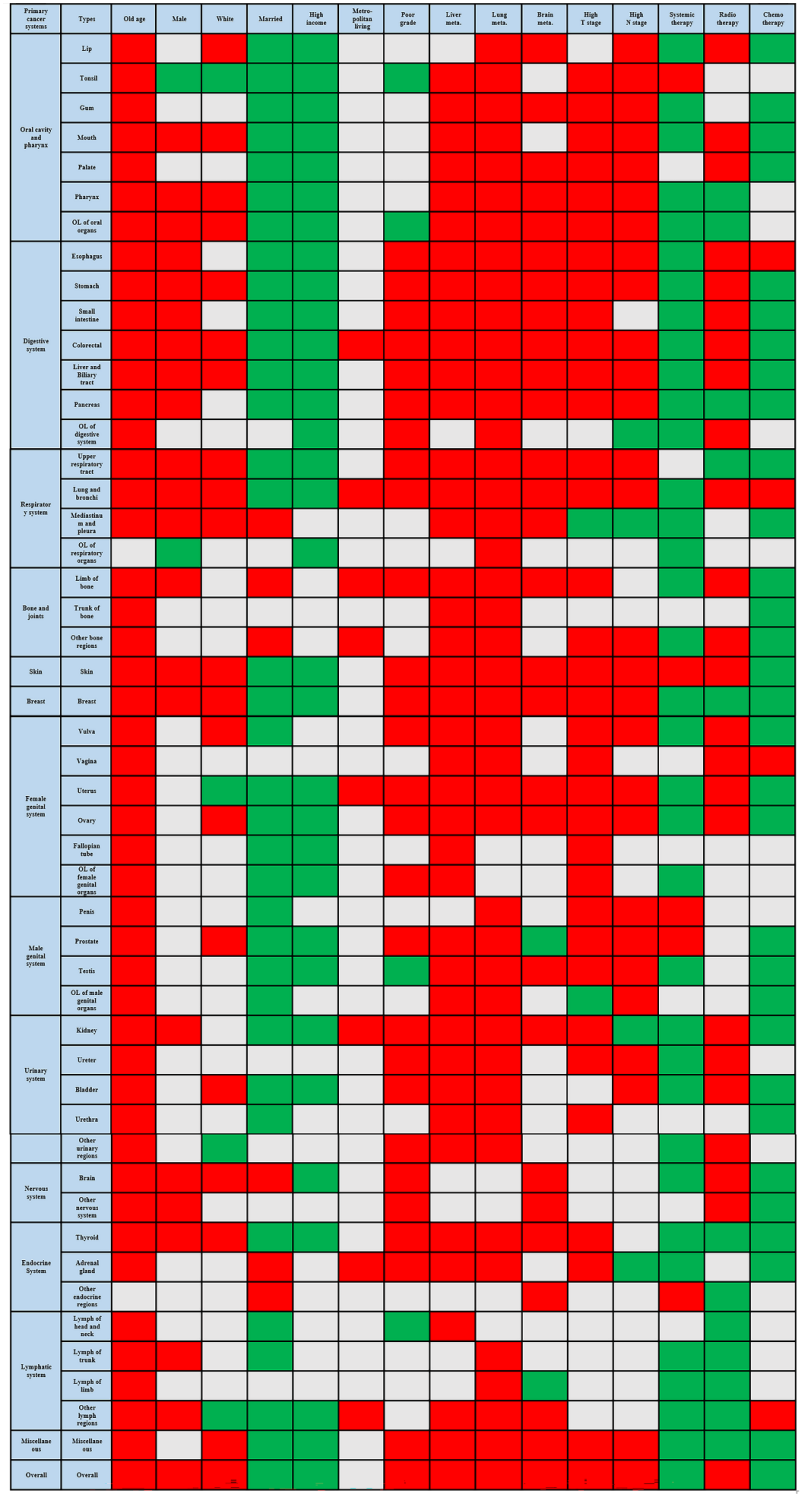
Risk factors related to the bone metastasis occurrence across various primary cancers.

The results of multivariable logistic regression analysis showed that old age (≥65), white race, poorer cancer grade, high T and N stages, synchronous lung, liver, and brain metastasis, and radiotherapy were risk factors for developing BM. On the other hand, married status, high income, systemic therapy, and chemotherapy were identified as protective factors against BM. The difference in living in metropolitan or non-metropolitan areas did not yield a significant impact on the occurrence of BM. As for the stratification of various primary cancer sites, the red area in the Figure 4 indicates the variables as risk factors for developing BM for a certain primary cancer, while the green area indicates variables as protective factors. The gray area represents no statistically significant difference.

### Stratified survival analysis

**Figure 5:**
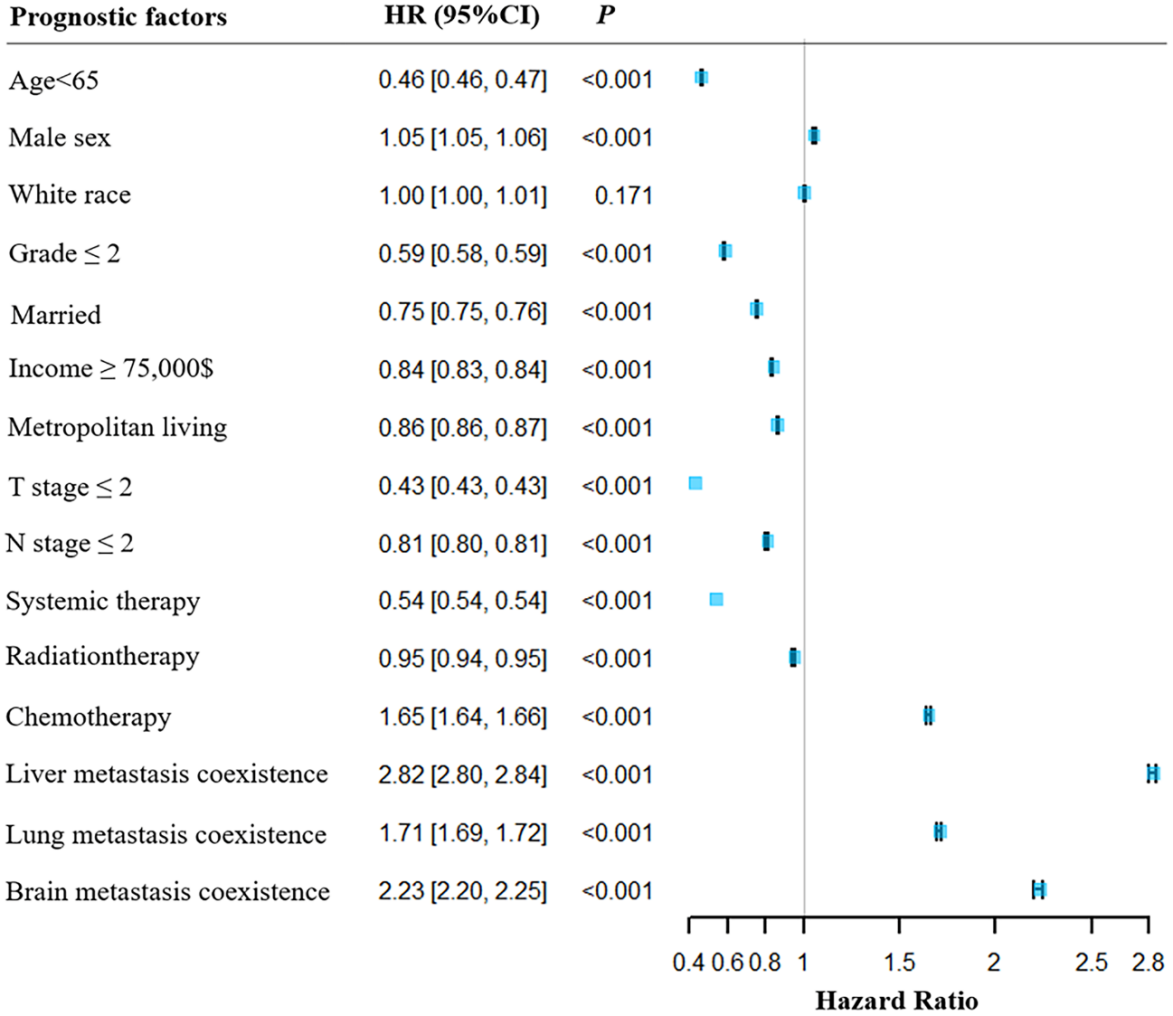
**Forest plot for the illustration the overall survival in patients with or without BM.** HR, hazard rate; CI, confidence interval.

The multivariable Cox regression analysis indicated that age < 65, primary cancer grade ≤ 2, married status, household income ≥ $75,000, T & N stages ≤ 2, and experiences with systemic and radiation therapy were associated with better survival rates for patients with BM. Conversely, male sex, chemotherapy experience, and coexisting liver, lung, and brain metastasis were associated with poorer survival rates for patients with BM. A forest plot was generated to illustrate the detailed hazard ratio (HR) and 95% confidence intervals (CIs) for each covariate (as shown in Figure 5).

According to Table 2, there were significant differences in overall survival rates and median survival time for patients with BM across different primary cancer sites. The cancers with the shortest median survival times were pancreatic cancer and cancer developed in OL of the digestive system, both with median survival times of 2 months. The primary cancer with the lowest overall survival rates has been identified as OL of the urinary organs with survival rates of 6.03%. Compared to patients without BM, the primary cancers that had the greatest negative impact on survival rates among patients with BM were lip cancer (HR = 34.99, 95%CI 8.64 - 141.70), thyroid cancer (HR = 22.27, 95%CI 20.14 - 24.63) and kidney cancer (HR = 19.84, 95%CI 19.53 - 20.17). After adjusting for confounding factors, only cancers that originated in the head and neck lymph regions did not show statistically significant differences.

**Table 2:** Survival analysis of different primary cancers with BM and hazard ratios of death comparing primary cancers with and without BM.

**Primary cancer sites**	**MST (m)**	**OS (%)**	**HR**	**95% CI**	***P***	**Adjusted *P***
Lip	6	33.33%	34.99	8.64 -141.70	<0.01	<0.01
Tonsil	6	14.03%	6.80	5.88 - 7.86	<0.01	<0.01
Gum	6	22.73%	3.90	2.41 - 6.29	<0.01	<0.01
Mouth	5	14.44%	5.02	4.01 - 6.27	<0.01	<0.01
Palate	11	25.00%	3.57	2.38 - 5.36	<0.01	<0.01
Pharynx	8	24.63%	2.94	2.64 - 3.27	<0.01	<0.01
OL of oral organs	8	20.05%	8.43	7.51 - 9.46	<0.01	<0.01
Esophagus	3	8.21%	2.80	2.67 - 2.93	<0.01	<0.01
Stomach	3	9.49%	3.38	3.23 - 3.54	<0.01	<0.01
Small intestine	6	35.75%	3.78	3.20 - 4.47	<0.01	<0.01
Colorectal	5	14.87%	6.31	6.08 - 6.55	<0.01	<0.01
Liver and biliary tract	3	9.40%	2.51	2.42 - 2.60	<0.01	<0.01
Pancreas	2	8.94%	1.99	1.91 - 2.07	<0.01	<0.01
OL of digestive systems	2	7.58%	1.54	1.41 - 1.69	<0.01	<0.01
Upper respiratory tract	6	19.94%	5.02	4.42 - 5.69	<0.01	<0.01
Lung and bronchi	4	11.52%	2.46	2.43 - 2.48	<0.01	<0.01
Mediastinum and pleura	7	22.93%	2.03	1.82 - 2.27	<0.01	<0.01
OL of respiratory organs	3	33.33%	1.76	0.35 - 8.77	0.49	<0.01
Limb of bone	13.5	39.63%	4.37	3.54 - 5.40	<0.01	<0.01
Trunk of bone	16	57.71%	2.04	1.77 - 2.35	<0.01	<0.01
Other bone regions	8	25.00%	11.83	8.3 - 20.71	<0.01	<0.01
Skin	5	22.54%	19.47	11.77 - 21.35	<0.01	<0.01
Breast	19	38.29%	9.78	0.46 - 0.48	<0.01	<0.01
Vulva	6	15.79%	7.34	5.51 - 9.77	<0.01	<0.01
Vagina	5	14.89%	4.43	3.22 - 6.10	<0.01	<0.01
Uterus	6	19.70%	10.93	10.36 - 11.54	<0.01	<0.01
Ovary	6	17.49%	4.04	3.63 - 4.49	<0.01	<0.01
Fallopian tube	20.5	35.71%	3.16	1.64 - 6.08	<0.01	<0.01
OL of female genital organs	3	10.29%	2.41	1.85 - 3.13	<0.01	<0.01
Penis	7	10.53%	7.74	4.77 - 12.55	<0.01	<0.01
Prostate	19	43.84%	13.03	12.77 -13.30	<0.01	<0.01
Testis	16	60.80%	10.67	8.48 - 13.42	<0.01	<0.01
OL of male genital organs	6	11.11%	11.7	5.70 - 24.02	<0.01	<0.01
Kidney	6	18.71%	19.84	19.53 - 20.17	<0.01	<0.01
Ureter	6	12.10%	4.48	3.67 - 5.48	<0.01	<0.01
Bladder	4	11.20%	10.98	10.43 - 11.55	<0.01	<0.01
Urethra	7	17.24%	6.04	3.93 - 9.29	<0.01	<0.01
OL of urinary organs	2	6.03%	5.51	4.38 - 6.93	<0.01	<0.01
Brain	15	50.63%	0.77	0.56 - 1.06	0.11	<0.01
Other nervous system	11	52.38%	4.13	2.19 - 7.75	<0.01	<0.01
Thyroid	14	45.23%	22.27	20.14 - 24.63	<0.01	<0.01
Adrenal gland	22	60.16%	0.91	0.78 - 1.07	0.27	<0.01
Other endocrine regions	38	55.56%	3.00	1.79 - 5.03	<0.01	<0.01
Lymph of head and neck	18	84.38%	1.84	1.10 - 3.09	0.02	0.08
Lymph of trunk	10	73.24%	1.48	1.13 - 1.94	<0.01	<0.01
Lymph of limb	14	73.03%	2.52	1.66 - 3.83	<0.01	<0.01
Other lymph regions	NR	73.87%	1.32	1.21 - 1.43	<0.01	<0.01
Miscellaneous	3	19.47%	4.16	4.00 - 4.34	<0.01	<0.01

OL, overlapping lesion; BM, bone metastasis; HR, hazard rate; CI, confidence interval; MST (m), median survival time (month); NR, not reached.

### Hierarchical cluster analysis

**Figure 6:**
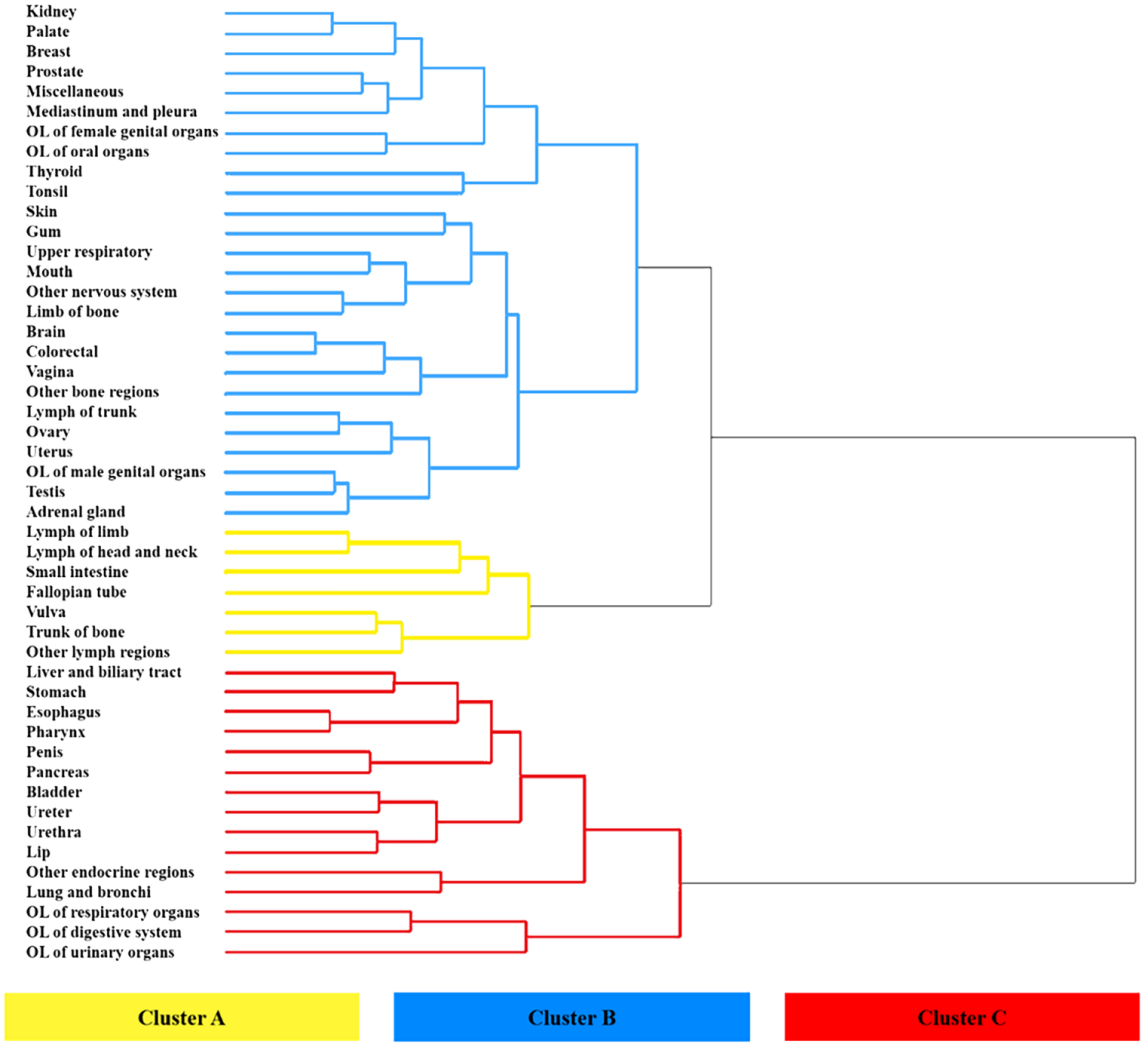
**Hierarchical clustering dendrogram of prognostic trend of 48 primary metastatic cancers.** Leaves marked in yellow, blue, and red were categorized as three different clusters.

**Figure 7:**
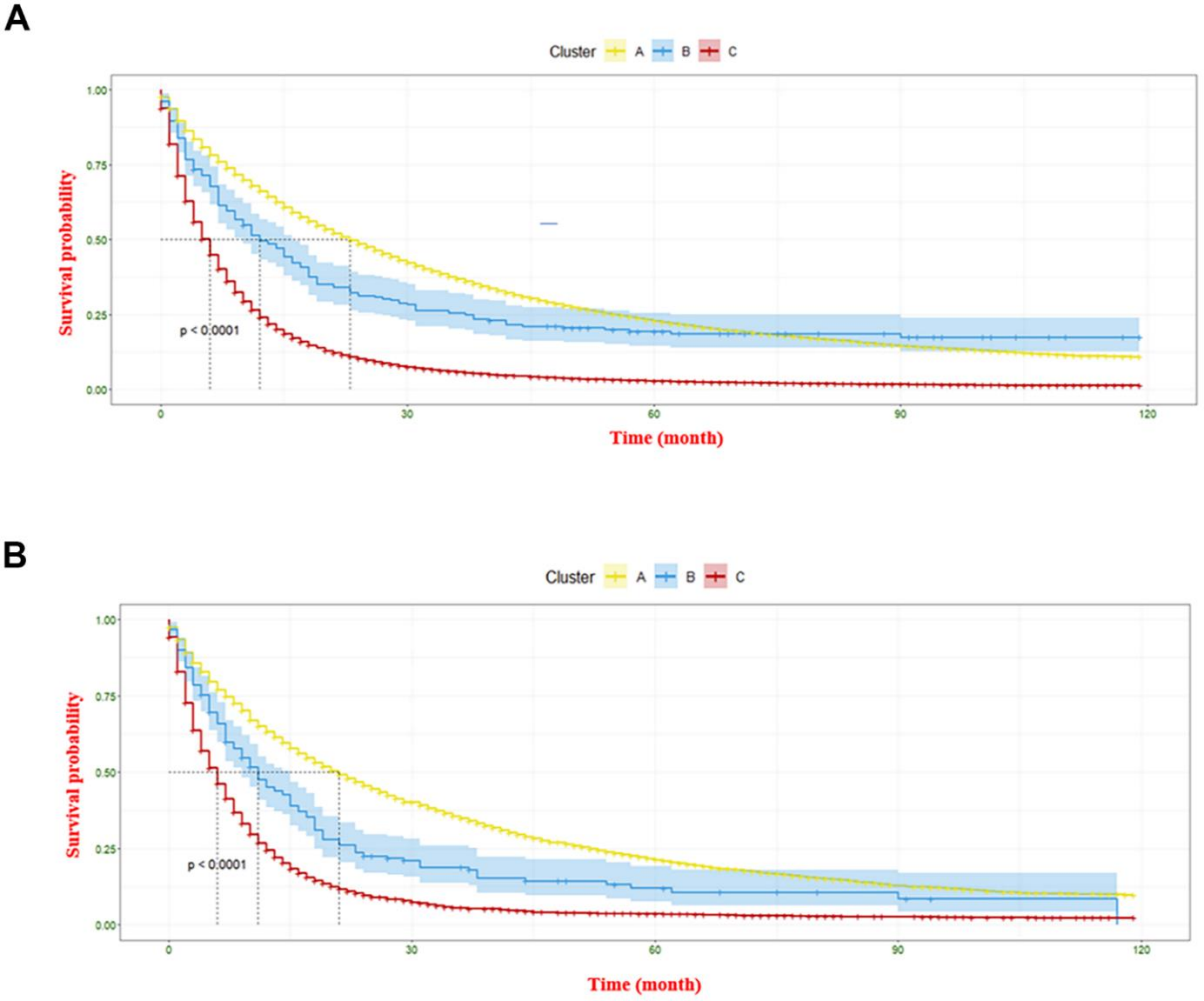
**The Kaplan-Meier curve displaying survival differences of three clusters.** (**A**) denotes the information gathered from the construction cohort, and (**B**) represents the information regarding the validation cohort.

The overall primary cancer sites (n = 48) in this study were classified into three clusters, namely A, B, and C, based on dendrogram from hierarchical clustering. The prognostic trend of each cluster can be clearly seen in Figure 6. Cluster A, which is marked in yellow in the figure, showed relatively best prognoses. This cluster comprises cancers that developed in the lymph nodes of the limb, head and neck, and other lymph regions, as well as those that occurred in the small intestine, fallopian tube, vulva, and bone trunk. Cluster C (marked in red) included cancers that originated from OL of respiratory, urinary, and digestive systems, lung and bronchus, lip, pancreas, esophagus cancers, etc. were observed the worst prognosis. The Kaplan-Meier curve were used to demonstrate the mean survival time for each category (as shown in Figure 7A). In the construction group, the mean survival time for clusters C, B, and A were 6.13 ± 0.16 months, 12.02 ± 0.09 months, and 22.95 ± 0.13 months, respectively. Similarly, in the validation group, the mean survival times were 5.92 ± 0.08 months, 11.01 ± 0.16 months, and 20.86 ± 0.07 months, respectively for cluster C, B and A (Figure 7B). Significant differences were observed in between the clusters in the construction as well as validation cohort (*P*<0.0001).

## DISCUSSION

The current study outlined the clinical characteristics and prevalence of BM from various primary cancers. We also provided information on overall survival and summarized the risk factors associated with BM. Of note, a reliable prognosis-based classification system was established by hierarchical cluster analysis. With the SEER database covering 28% of the U.S. population, the findings presented in this study were highly representative and can be leveraged to tailored bone metastatic monitoring and improved medical decision-making.

This study demonstrated from 2010 to 2019, that the number of cancer patients with BM has gradually increased but the number of involved deaths has decreased. Cancers originating from the adrenal gland, lung and bronchial sites were observed the highest prevalence of BM. The obtained result exhibited a degree of similarity to the findings of prior research studies. Huang et al. [10] found that the cancers with the highest prevalence of BM were other cancers in the urinary organs, adenocarcinoma of the lung, and other female genital cancers based on data from SEER from 2010 to 2016. However, we found that 25.03% (492 of 1966) of adrenal gland cancer cases had BM, which was inconsistent with previous findings. Previous studies have suggested that adrenal gland cancer is more likely to metastasize to the liver, lungs, and lymph nodes than to bone and brain [11, 12]. However, a recent study [13] has corroborated our findings. This study specifically examined the patterns of distant metastasis in adrenal gland cancer and discovered that the most prevalent sites of metastasis were the bone (42.3%), liver (38.4%), and then the lungs (30.5%). They also emphasized the importance of monitoring for adverse skeletal-related events (ASREs) in patients with adrenal gland cancer who have BM [12]. The study also investigated that compared to other cancers such as lung and renal cancer, adrenal gland cancer patients with BM had a high risk of ASREs, which included spinal cord compression, fractures, and hypercalcemia. In fact, over one-third of patients were diagnosed with BM due to the earliest symptoms or signs of ASREs [11, 14]. Similarly, patients with lung and bronchus cancers had also a high prevalence of BM in this study. It has been demonstrated that around 80% of lung cancer patients that with BM frequently experience pain as their first symptom [15]. The spine has been revealed as the most frequent site for lung cancer metastasis, with the ribs and pelvis being the subsequent common sites [16]. Strong associations with increased risk of death and ASREs were observed in bone metastatic lung cancer [17]. Therefore, it is crucial to raise awareness of BM originated from adrenal gland, lung and bronchus sites and to improve related early diagnosis (e.g., using 18F-FDG PET, liquid biopsy) [18, 19].

Cancers that occur in the OL of digestive system, e.g., a carcinoma happened in junction region of the stomach and small intestine, carry a higher risk of BM. Accurate cancer diagnosis and classification posed a challenge at times. Since it was frequently observed that cancer occurred at the junction of two or more anatomical regions, making it challenging to identify the specific origin of the cancer. This could either be because the cancer originated from this overlapping area, or surrounding infiltration of cancer cells was already evident at diagnosis. A study [20] also found that esophageal cancer occurring in the overlapping lesion had a higher chance of developing brain metastasis. Siotos et al. [21] identified that breast cancer originating from overlapping lesions had higher odds of (OR = 1.58; 95% CI, 1.3-1.83) positive axillary lymph nodes and posed higher risk of death (HR = 1.28; 95% CI, 1.05-1.55). Currently, to the best of our knowledge, no literature has investigated the prevalence of BM in cancers that develop in the overlapping lesion of the digestive system.

This study identified factors such as age ≥ 65, white race, poorer cancer grade, high T and N stages, and synchronous lung, liver, and brain metastasis were positively related to the occurrence of BM. Although no studies have specifically examined risk factors for pan-cancer-related BM, numerous publications have analyzed relevant risk factors when BM occurs in specific cancer types, such as breast cancer, lung cancer, and kidney cancer, and many of their results are consistent with this study [5, 20, 22, 23]. Survival analysis revealed that age < 65, primary cancer grade ≤ 2, married status, household income ≥ $75,000, T & N stages ≤ 2, and with systemic- as well as radiation-therapy experiences were associated with better survival for patients with BM, while male gender, chemotherapy, and coexistences with liver, lung, and brain metastasis showed poorer survival rates. Surprisingly, stratified analysis showed that the survival rates of cancers occurred in urinary, digestive overlapping regions and esophagus were the lowest among all 48 primary cancers when accompanied by BM. This finding highlights the importance of early identification of cancers originating from overlapping areas of the urinary and digestive systems, including the esophagus, when assessing the prognosis of patients with BM. The literature either agrees or disagrees with this finding. The survival rate for lung cancer with BM is low, with a typical survival time of less than one year [23, 24]. There were studies found that bladder cancer with BM may have a 5-year survival rate as low as 4.6% to 6.8% [25, 26]. However, these articles did not discuss cancers that were developed in the overlapping lesion. The reason may be attributed to the use of different classification systems (for example WHO histological classification of cancer) or the generalization of cancers with overlapping lesion into a larger system category. In addition, although BM is relatively uncommon in patients with esophageal cancer, it often appears in the later stages of the disease. Despite being less common than liver, lung, or brain metastases, patients with esophageal cancer who developed BM tend to have poorer prognoses, with a median survival time of 5 months and a 5-year overall survival rate of 5% [27]. This result was similar to that of the current study.

A prognosis-based classification system for pan-cancers with BM was established in this study. It is worth noting that cancer patients categorized under cluster C should prioritize prompt treatment planning due to their significantly shortened survival time after the emergence of bone metastasis. Traditional anatomy-based classification systems (such as respiratory and digestive systems) cannot provide accurate prognosis prediction information for metastatic patients with BM [28]. Further, incorrectly classifying cancers that occur in overlapping lesions may result in a significant bias in prognosis analysis. The proposed classification system not only provides survival information for guiding physicians to select personalized treatment plans for metastatic patients with BM but also assists medical staff in optimizing the allocation of health care resources. In the current literature, hierarchical clustering has been also used to classify various patients with pan-metastatic cancers. Zhang and his teammates [29] used hierarchical clustering analysis to construct a classification system based on survival and prognostic factors, thus dividing stage IV metastatic patients into three categories. This approach helps provide a novel strategy for guiding the prediction of survival and personalized treatment for stage IV metastatic patients. Another study identified three subtypes of gastric cancer based on gene expression patterns and cell composition by using hierarchical clustering analysis [30]. It was determined that subtypes associated with the highest mortality showed high stromal scores and infiltration of fibroblasts and endothelial cells, while subtypes with the best prognosis had high infiltration of various immune cells. These results provide valuable insights for personalized treatment of gastric cancer patients.

There were some limitations in this study. First, since our study was retrospective and relied solely on data from the SEER database, there is a possibility of selection bias in the patient selection process. Second, some important variables such as menopausal status, tumor size of primary cancer and alkaline phosphatase (ALP) level were not contained in the current SEER database, which may have led to inevitable biases. Third, since the study is a pan-cancer research, it is not possible to specifically analyze the occurrence and prognosis of BM in a particular type of cancer (i.e. examining whether breast cancer developed in nipple is positively related to BM occurrence, or how serum ALP concentration contributed to the prognosis of clear cell renal cell carcinoma patients with BM). Fourth, the cancer classification system developed in the study needs further multicenter validation with larger datasets to ensure its feasibility and generalizability. Fifth, patients with BM are more likely to have other regions metastasis due to its spread mechanism, which may confound the survival analysis. To address this issue, competing risk regression is a promising technique that may be used in the future study to minimize this impact.

## CONCLUSIONS

This study analyzed the clinical features, incidence, risk factors, and prognostic factors in pan-cancer patients with BM occurrence. A prognosis-based classification system was established for the first time using hierarchical clustering to categorize various primary cancers with BM. This system is expected to provide survival information to help physicians select personalized treatment plans for metastatic patients with BM who have poor prognoses.

## MATERIALS AND METHODS

### Data retrieval

The data used in this study were obtained from the SEER database, which was recognized as one of the world’s most prominent data sources for the follow-up of cancer patients, offering dependable data for clinical research.

### Study population

The informed consents of involved patients were not required due to the public nature of the database. Patients diagnosed with cancer from 2010 to 2019 based on version 7 of the American Joint Committee on Cancer (AJCC, www.cancerstaging.org) and containing clinical information (age, sex, race, tumor grade, marital status, income, living area, cancer T & N stage, metastasis conditions, systemic, radio- and chemotherapy) were included in this study. Patients who were 1) below 18 years old or 2) received a BM diagnosis at their autopsy or on their death certificate were excluded. All information was extracted using SEER*Stat version 8.4.1 (Inc., Calverton, MD, USA). Cases with primary sites in bone and joint were excluded. A flow chart of the patient selection was presented in the Supplementary Figure 1.

### Statistical analysis

Chi-square test and rank-sum test were used for categorical and numerical variables, respectively. R software (version 4.2.2) was used for performing statistical analyses and visualization. A multivariable logistic regression incorporating all factors was performed to determine the associated factors to BM occurrence. A Cox proportional hazards model was conducted to screen possible factors linked to overall survival in patients with BM. Statistical significance was set at *P* < 0.05.

A hierarchical clustering analysis using Euclidean distance based on survival time was conducted [31], the involved code of R was demonstrated in Supplementary Figure 2. The optimal number of clusters was determined using a dendrogram that illustrates the merging or splitting sequences of the clusters [32]. Patients diagnosed between 2015 and 2019 were included as the construction cohort, while the validation cohort included those who were diagnosed between 2010 and 2014. The Kaplan-Meier estimate was performed to determine the prognosis of the different cancer categories, and differences of each category were identified with the log-rank test.

### Data availability statement

In this study, publicly available datasets were utilized for analysis. The data used in the study can be accessed at the following website: https://seer.cancer.gov/data/.

## Supplementary Material

Supplementary Figures
