# Supplementary material for: Prevalence and risk factors of bone metastasis and the development of bone metastatic prognostic classification system: a pan-cancer population study
Source: Aging (Albany NY). 2023 Nov 19;15(22):13134–49. doi: 10.18632/aging.205224 (PMC10713404; doi:10.18632/aging.205224)
Supplement: Supplementary Figures [file aging-15-205224-s001.pdf]

SUPPLEMENTARY FIGURES

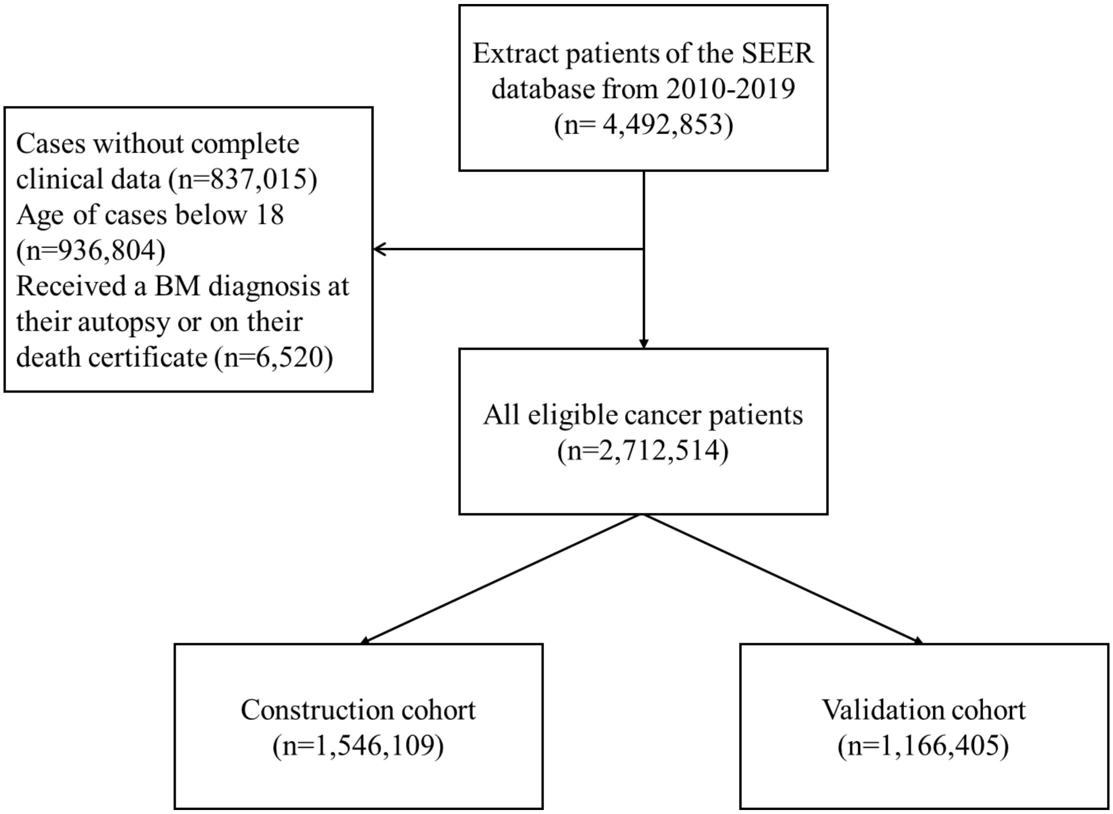

Supplementary Figure 1. A flow chart of the patient selection for the construction and validation cohorts in cancer patients with bone metastasis (BM).

```
HCdata<- read.csv("C:\\data.csv", header=T, row.names = 1)
head(HCdata)
dim(HCdata)
hc<- hclust(dist(HCdata[,1:17],method = "euclidean"))
library(ggdendro)
library(cluster)
df <- dendro_data(hc,type="rectangle")
df1 <- df$segments
df2 <- df$labels
head(df1)
head(df2)
library(ggplot2)
ggplot()+
  theme_void() +
  coord_flip()+
  geom_segment(data=df1,aes(x=x,y=y,xend=xend,yend=yend))+
  geom_text(data=df2,aes(x=x,y=y-4,label=label,size=3,hjust=0, vjust=0.5))
```

Supplementary Figure 2. The hierarchical clustering code and visualization used in R.
